# Patterns and predictors of gestational weight gain in Addis Ababa, Central Ethiopia: a prospective cohort study

**DOI:** 10.1186/s12978-021-01202-y

**Published:** 2021-07-28

**Authors:** Fekede Asefa, Allison Cummins, Yadeta Dessie, Maralyn Foureur, Andrew Hayen

**Affiliations:** 1 School of Public Health, College of Health and Medical Sciences, Haramaya University, Harar, Ethiopia; 2 Centre for Midwifery, Child and Family Health, Faculty of Health, University of Technology Sydney, Ultimo, Sydney, NSW Australia; 3 School of Public Health, Faculty of Health, University of Technology Sydney, Ultimo, Sydney, NSW Australia; 4 Hunter New England Health, Nursing and Midwifery Research Centre, University of Newcastle, Newcastle , NSW Australia

**Keywords:** Gestational weight gain, Predictors, Body mass index, Ethiopia

## Abstract

**Introduction:**

Gaining excessive or inadequate gestational weight is associated with many adverse maternal and fetal outcomes. Inadequate gestational weight gain (GWG) increases the risk of fetal growth restriction, pre-term birth, and low birth weight. It is a public health concern in sub-Saharan Africa. The aim of this study was to assess the patterns and predictors of GWG in Addis Ababa, Ethiopia.

**Methods:**

We conducted a prospective cohort study among pregnant women who attended antenatal care in health centres in Addis Ababa, from January to September 2019. Data were collected by a structured questionnaire and checklists and analysed using Stata version-14. Weight at or before 16 weeks gestation was used as a proxy for pre-pregnancy weight. Women’s height and baseline weight were measured by data collectors, and we obtained weight at the end of the 24th and 36th weeks of gestation from women’s medical records. GWG was categorized as inadequate, adequate and excessive based on the United States Institute of Medicine criteria. Predictors of GWG were identified using multinomial logistic regression.

**Results:**

A total of 395 pregnant women were enrolled in the study. GWG was assessed for 369 (93%) women. The median GWG was 8.7 kg with inter quartile ranges (25th, 75th percentiles) of 7.0 kg and 11.6 kg. More than two-third of the participants, 248 (67.2% [95% CI: 62.2, 72.0%]), gained inadequate weight; 103 (27.9% [95% CI: 23.4, 32.8%]) gained adequate weight; and 18 (4.9% [95% CI: 2.9%, 7.6%]) gained excessive weight. Three quarters (75%) of underweight women gained inadequate gestational weight, whereas 43% of overweight or obese women gained inadequate gestational weight. Being underweight (AOR = 3.30 [95% CI: 1.32, 8.24]) or normal weight (AOR = 2.68 [95% CI: 1.37, 5.24]) before pregnancy increased the odds of gaining inadequate gestational weight compared to overweight or obese women. Not having paid employment was associated with higher odds of gaining inadequate gestational weight compared to women employed outside the home (AOR = 2.17 [95% CI: 1.16, 4.07]).

**Conclusions:**

Most pregnant women in Addis Ababa gain inadequate gestational weight. In particular, three quarters of underweight women gained inadequate gestational weight. Being underweight, normal weight or having no paid employment were associated with higher odds of inadequate GWG. Promoting adequate GWG in Addis Ababa among underweight and normal weight women may be an important public health initiative.

**Supplementary Information:**

The online version contains supplementary material available at 10.1186/s12978-021-01202-y.

## Plain language summary

Pregnant women are expected to gain weight due to various biological changes. Gaining inadequate or excessive weight during pregnancy poses a health risk to the mother and baby. Inadequate weight gain is a public health concern in low-income countries, including Ethiopia. This study assessed patterns and predictors of weight gain during pregnancy in Addis Ababa, Central Ethiopia. The study was conducted from January to September 2019. Women attending public health facilities were followed from before or at 16 weeks of gestation until 36 weeks of pregnancy. A total of 395 pregnant women enrolled in the study. Weight gain was assessed for 369 (93%) women. More than two-third of the participants (67.2%) gained inadequate weight during pregnancy. Three quarters (75%) of underweight women gained inadequate weight, whereas 43% of overweight or obese women gained inadequate weight during pregnancy. Promoting adequate weight gain during pregnancy for underweight and normal weight women in Addis Ababa is an important public health initiative.

**Supplementary Information:**

The online version contains supplementary material available at 10.1186/s12978-021-01202-y.

## Introduction

Gestational weight gain (GWG) is defined as the amount of weight gain from conception until the birth of the baby. The recommended amount of GWG varies based on pre-pregnancy body mass index (BMI) of the women. According to the 2009 United State Institute of Medicine (IOM) recommendations women with BMI ≤ 18.5 kg/m^2^ are recommended to gain 12.5–18 kg; women with BMI 18.6–24.9 kg/m^2^ are recommended to gain 11.5–16 kg; women with BMI 25.0 to 29.9 kg/m^2^ are recommended to gain 7–11.5 kg; and women with BMI ≥ 30.0 kg/m^2^ are recommended to gain 5–9 kg [1].

Most studies have reported that fewer than a third of pregnant women gain adequate gestational weight [2–11]. The majority of women in high income countries such as the United States (73%) [2] and Canada (71%) [6] gain an excessive amount of gestational weight. In contrast, inadequate GWG is a major public health concern in low-income Sub-Saharan African countries [12, 13] where studies showed that 69% of pregnant women from eastern Ethiopia, Harar [5], and 62.7% of pregnant women from low income settings such as urban Uganda, Kampala [14] gained inadequate gestational weight. Pregnant women who gain inadequate weight are at a higher risk of bearing a baby with low birth weight (LBW) and pre-term birth [3, 15–17]. In Ethiopia, the prevalence of LBW (17%) [18] and preterm birth (26%) [19] are among the highest worldwide. On the other hand, women who gained excessive gestational weight are at a higher risk of developing gestational hypertension [2, 3], gestational diabetes [2], caesarean birth [20], high postpartum weight retention [4], and long term obesity [21].

The amount of GWG is associated with numerous factors. These include health system related factors (antenatal care including advice and follow-up from health care providers) [22–24], and women’s own related factors (physiological factors, psychological, and behavioural factors) [1, 25–27]. Women’s age [5], pre-pregnancy maternal weight [5, 28, 29], multi-parity [6, 7], women’s awareness of healthy eating and dietary diversity [30], attending antenatal care [5], and engaging in physical activity [5, 31] are all related to the amount of GWG.

Studies from high-income countries report that household food insecurity is associated with excessive GWG [32, 33]. However, this needs further investigation in low-income countries where women suffer from nutritional deficiencies in households with food insecurity [34, 35]. Furthermore, household food insecurity is strongly associated with low nutritional status such as being underweight [36, 37], decreased mid-upper arm circumference (MUAC) [34, 37], and anaemia [38]. Though some authors [32, 33] have argued that food insecurity causes excessive GWG leading to maternal depression, it is unclear whether food insecurity causes maternal depression or maternal depression causes food insecurity [39].

Having a history of intimate partner violence during pregnancy is associated with gaining less weight during pregnancy [40, 41]. Despite the high levels of intimate partner violence in Ethiopia (emotional violence 24.0%, physical violence 24.9% and sexual violence 11.1%) [42], its effect on GWG is not well studied.

Few studies have been conducted in Ethiopia focusing on GWG [5, 43, 44]. Published studies have retrospectively assessed GWG and prospective studies are recommended [13]. Retrospective studies have not addressed some important predictor variables such as physical activity, food insecurity, perinatal depression, and intimate partner violence during pregnancy. The aim of the current study was to assess the patterns and predictors of GWG in Addis Ababa, central Ethiopia.

## Methods

### Study setting

This study was conducted in Addis Ababa, which is the capital and largest city in Ethiopia. In the city, there are 42 hospitals (11 government, 6 non-government organisations, and 25 private), 97 Health Centres, and 361 clinics that provide medical care including maternal health care [45, 46]. Around 97% of pregnant women in Addis Ababa receive antenatal care (ANC) from skilled care providers such as doctors, nurses or midwives, at least once [42], of which 90% receive at least four ANC contacts [47].

### Study design

A prospective cohort study design was employed from January 2019 to September 2019.

### Sample size determination

We calculated the sample size using Open Epi Version 2.3 considering both the single proportion formula (to assess the proportion of GWG) and the double proportion formula (to assess predictors of GWG). The larger sample size was achieved by using the single proportion formula considering the proportion of women with inadequate gestational weight from a study conducted in Harar, Ethiopia (*p* = 0.69) [5], a half-width of confidence 5%, an alpha value of 0.05, and 20% loss to follow-up. The final sample size was 395.

### Participants and sampling procedure

The women were selected from nine health centres. The health centres were selected based on the number of ANC visits and geographic location in the city. Women who met the inclusion criteria were consecutively selected from each health facility until the required sample size was met.

We invited all pregnant women in their first trimester (before 16 weeks gestation) who came to the selected health centres for antenatal care (Additional file 1: Table S1). Antenatal clinic staff who provided the antenatal care facilitated the participant selection process. They also assisted in setting appointment dates for the follow-up data collection (at the end of the 24th and 36th weeks of gestation). Women with a twin-pregnancy or with co-morbidities such as diabetes and hypertension were excluded from the study.

### Measurements

We collected data through face-to-face interviews and review of health records. We used a range of tools to collect data on socio-demographic characteristics, dietary diversity and food security, intimate partner violence, physical activity and depression related symptoms. Variables such as gestational age (ultrasound result), blood pressure, random blood sugar, anaemia status, and HIV status were obtained from medical records of the women. Principal component analysis was employed to compute a wealth index [48] from a set of household assets questions such as electricity, refrigerator, table, chair, watch, phone, bed with mattress, electric mitad (an Ethiopian oven made up of clay and metal), car, house, improved water, and improved toilet, which were adapted from the Ethiopian demographic and health survey [42].

Gestational age was estimated by the last menstrual period and verified by ultrasound which was a routine practice of the health facilities. The gestational age of our study participants ranged from four to 16 weeks (8.9% were between four and seven weeks of gestation; 41.1% were between eight and 12 weeks of gestation; and 50% were between 13 and 16 weeks of gestation). The height of the women was measured when barefoot using a height measuring board in a standing position and recorded to the nearest 0.1 cm. The maternal weight was measured by a digital weight scale with minimum clothing and the reading was recorded to the nearest 100 g. We asked women if they knew their pre-pregnancy weight, however only 172 (43.5%) of the participants were aware of their pre-pregnancy weight. Therefore, we used weight at or before 16 weeks as a proxy for pre-pregnancy weight in all women. Women’s height and baseline weight were measured by data collectors, while weight at the end of 24th and 36th weeks of gestation was collected from women’s medical records. Body Mass Index (BMI) was calculated by dividing weight by height, squared. The women’s BMI at or before 16 weeks of gestation (for those whose ages were ≥ 20 years old) was categorized into four categories based on the World Health Organization BMI cut-off points as underweight (BMI ≤ 18.5 kg/m^2^); normal weight (18.6 to 24.9 kg/m^2)^; overweight (25.0 to 29.9 kg/m^2)^; and obese (≥ 30.0 kg/m^2^). BMI-for-age (at or before 16 weeks of gestation) was calculated for adolescent women (women aged 18 and 19 years old); and BMI was categorized using WHO reference cut-off points as thin (Z-score < -2 standard deviation (SD)), normal (-2 SD ≤ Z-score ≤  + 1SD), overweight (+ 1SD < Z-score ≤  + 2SD) and obese (Z-score >  + 2SD). Total weight gain was calculated by subtracting the pre-pregnancy weight from their weight at the 4th antenatal care visit (at the end of 36 weeks of gestation). It was categorized as inadequate, adequate and excessive according to the IOM classification. Mid upper arm circumference (MUAC) was measured using an adult MUAC non-stretchable measuring tape and the reading was taken to the nearest 0.1 cm. A MUAC measurement below 23 cm was categorised as low (or wasting) and above 23 cm was categorized as normal.

Dietary diversity of the women was assessed using a minimum dietary diversity-women (MDD-W) set from the Food and Agricultural Organisation (FAO) and USAID’s Food and Nutrition Technical Assistance III Project (FANTA) [49]. The food groups assessed in MDD-W include: grains, white roots, tubers and plantains; pulses; nuts and seeds; dairy; meat, poultry and fish; eggs; vegetables; other vitamin A-rich fruits and vegetables; other vegetables; and other fruits. The MDD-W is a dichotomous indicator of whether or not women have consumed at least five out of ten defined food groups the previous day or night. The proportion of women who reach this minimum can be used as a proxy indicator for higher micronutrient adequacy.

Household food insecurity was assessed using the Household Food Insecurity Access Scale (HFIAS). In each domain of the HFIAS questions ask about anxiety and uncertainty; insufficient quality; and insufficient food intake and any physical consequences, with a recall period of four weeks (30 days)[50].

Women's physical activity level was measured using the International Physical Activity Questionnaire (IPAQ-long form). The IPAQ assesses physical activity across a range of different domains including recreation-time, housework, being employed and transportation related physical activities. Each domain assesses walking, moderate and vigorous physical activities over a seven day period. Women were asked if they had completed these activities continuously for at least 10 min. Responses to IPAQ questions on the frequency and duration of physical activity were converted to the metabolic equivalent task per minute (MET-minutes) [51]. A MET is the ratio of specific physical activity metabolic rates to the resting metabolic rate, with one MET defined as the energy needed by an individual while at complete rest, which is equivalent to l kilocalorie per kilogram per hour [52].

The level of physical activity for each woman was categorized as;

- High—the woman accumulated at least 1500 MET-minutes per week from vigorous-intensity activity on at least 3 days; or accumulated at least 3000 MET-minutes per week on seven or more days of any combination of walking, moderate- or vigorous-intensity activities;
- Moderate—the woman engaged in three or more days of vigorous-intensity activity of at least 20 min per day; five or more days of moderate-intensity activity and/or walking of at least 30 min per day; or five or more days of any combination of walking, moderate-intensity or vigorous intensity activities achieving a minimum of at least 600 MET-minute per week,
- Low—the woman reported no activity or some, but not enough to meet the high and moderate categories [51].

Perinatal depression symptoms were measured using the Edinburgh postnatal depression scale (EPDS) [53], which is a ten-item questionnaire. It has been validated and used by many studies for detecting perinatal depression in Ethiopia [54–57].

Intimate partner violence was measured with a questionnaire used by the WHO multi-country study on women’s health and domestic violence [58]. It includes physical violence, sexual violence and emotional abuse by intimate partners. This questionnaire has also been used in the Ethiopian Demographic and Health Survey (EDHS) 2016 [42], making the survey suitable to use in the current study setting.

### Statistical analysis

Data were entered into CSPro version 7.1, and exported to STATA (V.14, Stata Corp, 2015) for analysis. Frequencies and proportions were estimated to describe the variables. BMI-for-age was calculated for adolescent pregnant women using WHO AnthroPlus software. We conducted bivariable and multivariable analyses using a multinomial logistic regression model, because the outcome variable (i.e., GWG) consisted of three categories (inadequate, adequate and excessive GWG). Pregnant women with inadequate or excessive GWG were compared to women with adequate GWG (reference category). Variables with *P*-value < 0.25 in the bivariate analysis were included in the multivariable analyses. The variables in the multivariable analyses included educational status, wealth index, occupational status, BMI, MUAC, perinatal depression and asking permission from partners for health care seeking. Crude odds ratios (COR) and adjusted odds ratios (AOR) were calculated to determine the association between the explanatory variables and GWG.

## Results

**Fig. 1:**
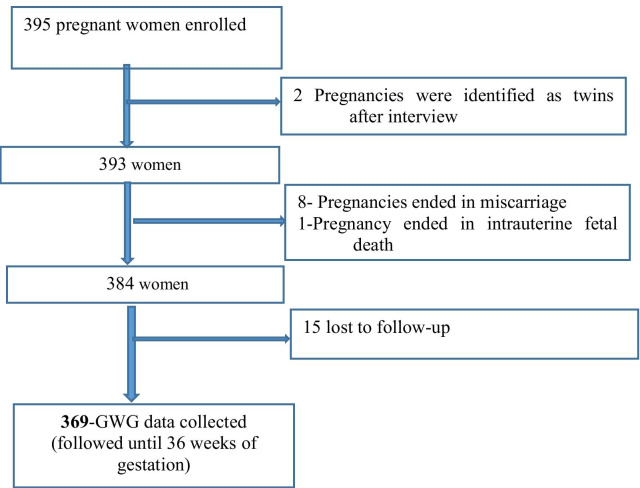
Flow chart of study participants’ follow-up in Addis Ababa, Ethiopia, 2019

We enrolled 395 women into the study. Of these women, we recorded GWG for 369 women with a follow-up rate of 93.4% (Fig. 1).

### Socio-demographic characteristics

The mean age of the women was 25.3 (standard deviation = 3.9) years, and 80.5% were between 20 and 29 years of age. Most of the respondents (96.5%) were married; 9.7% never attended formal education; and 50.6% were not employed outside of the home. All were urban residents. Seventy two (72%) percent of the respondents had an estimated income of < 200USD per month. Ninety percent (90%) of the pregnancies were intended; almost half (49.6%) of the women were primigravida (Table 1).

**Table 1:** Baseline demographic characteristics of the study participants in Addis Ababa, Ethiopia, 2019 (n = 395)

Variable	Frequency	Percentage
Age		
Less than 20 years	20	5.0
20–29 years	318	80.5
30–39 years	56	14.3
Greater than 40 years	1	0.2
Marital status (395)		
Never married	14	3.5
Married	381	96.5
Educational status (394)		
No formal education	38	9.7
Primary education (1–8)	162	41.1
Secondary education (9–12)	112	28.4
Tertiary education	82	20.8
Partners’ educational status (395)		
No formal education	32	8.1
Primary education (1–8)	99	25.1
Secondary education (9–12)	147	37.2
Tertiary education	117	26.6
Occupational status		
No paid employment (home duties)	200	50.6
Government/private employee	126	31.9
Merchant	39	9.9
Student	8	2.0
Daily labourer	12	3.0
Other*	10	2.6
Estimated average monthly income (364)		
≤ $ 200 USD	263	72.3
> $ 200 USD	101	27.7
Type of pregnancy (388)		
Intended	351	90.5
Unintended	37	9.5
Gravidity (395)		
Primigravida	196	49.6
Multigravida	199	50.4

*farmer, waitress, selling coffee and tea on the street, freelance researcher

### BMI and GWG of the study participants

The mean BMI of the respondents (with age ≥ 20 years old) at or before 16 weeks of gestation (which was a proxy for pre-pregnancy weight) was 21.83 (SD = 3.28 kg/m2); 18.1% had a BMI of ≤ 18.5 kg/m^2^; 63.7% had a BMI between 18.5 and 24.9 kg/m^2^; 17.3% had a BMI between 25 and 29.9 kg/m^2^; and 0.9% had a BMI ≥ 30 kg/m^2^. According to the BMI-for-age of adolescent pregnant women (aged 18 and 19 years old): 5.0% were thin; 80.0% were normal weight; 10.0% were overweight; and 5.0% were obese. Women were asked if they knew their pre-pregnancy weight, however only 172 (43.5%) of the participants were aware of their pre-pregnancy weight. The median GWG was 8.7 kg with inter quartile ranges (IQR) (25th, 75th percentiles) of 7 kg and 11.6 kg; underweight women gained a median weight of 10.0 kg with IQR of 7.6 kg and 12.5 kg while overweight or obese women gained a median weight of 7.0 kg with IQR of 5.0 kg and 8.2 kg (Table 2). Of 369 women for whom GWG was assessed, 67.2% (95% CI: 62.2, 72.0%) gained inadequate gestational weight; 27.9% (95% CI: 23.4, 32.8%) gained adequate gestational weight; and 4.9% (95% CI: 2.9, 7.6%) gained excessive gestational weight. Three quarters (75%) of underweight women gained inadequate gestational weight whereas 43% of overweight or obese women gained inadequate gestational weight.

**Table 2:** Patterns of gestational weight gain by specific body mass index at specific trimester in Addis Ababa, Ethiopia, 2019

Weight status	Second trimester GWG (Median and IQR (25th, 75th percentiles) (n = 328)	Third trimester GWG (Median and IQR (25th, 75th percentiles) (n = 324)	Total GWG (Median and IQR (25th,75th percentiles) (n = 369)
Underweight women	5 kg (3.5, 6.0)	4.5 kg (3.0, 6.6)	10 (7.6, 12.5)
Normal weight women	4 kg (3.0, 5.7)	5 kg (3.0, 6.1)	9 kg (7.0, 12.0)
Overweight or obese women	3.9 kg (1.0, 5.7)	4 kg (2.0, 5.0)	7 kg (5.0, 8.2)
Total	4 kg (2.9, 6.0)	4.5 kg (3.0, 6.0)	8.7 kg (7.0, 11.6)

### Dietary diversity, food security, and anaemia status

Within the previous 24 hours before the interview, 95.6% of the participants consumed foods prepared from grains; 28.4% consumed meat and meat product; and 6.4% consumed fish and fish products. Women’s dietary diversity score varied from 1 to 10, with 17.2% having a low dietary diversity score. Thirteen percent of the study participants were living in food-insecure households; 3% had anaemia (2.4% had mild anaemia; 0.3% each had moderate and severe anaemia); and 18% had low MUAC value (< 23 cm) (Table 3).

**Table 3:** Dietary diversity, food security, anaemia status of the study participants in Addis Ababa, Ethiopia, 2019 (n = 395)

Variable	Frequency	Percentage
Food group consumed in the last 24 h		
Foods from grains	377	95.7
White roots, tubers and plantains	273	69.3
Pulses (bean, peas and lentils)	264	67.0
Milk and milk products	138	35.0
Meat and meat product	112	28.4
Any fresh or dried or shellfish	25	6.4
Eggs	89	22.6
Vegetable	229	58.1
Fruits	229	58.1
Any foods made with oil or fat	341	86.6
Minimum dietary diversity		
Low dietary diversity	68	17.2
High dietary diversity	327	82.8
Food Security (391)		
Food secure	340	87.0
Mildly food insecure	19	4.9
Moderately food insecure	26	6.6
Severely food insecure	6	1.5
MUAC (387)		
Low MUAC (< 23 cm)	70	18.1
High MUAC (≥ 23 cm)	317	81.9
Had anaemia (385)		
Yes	11	3.0
No	374	97.0

### Respondents’ physical activity status

**Fig. 2:**
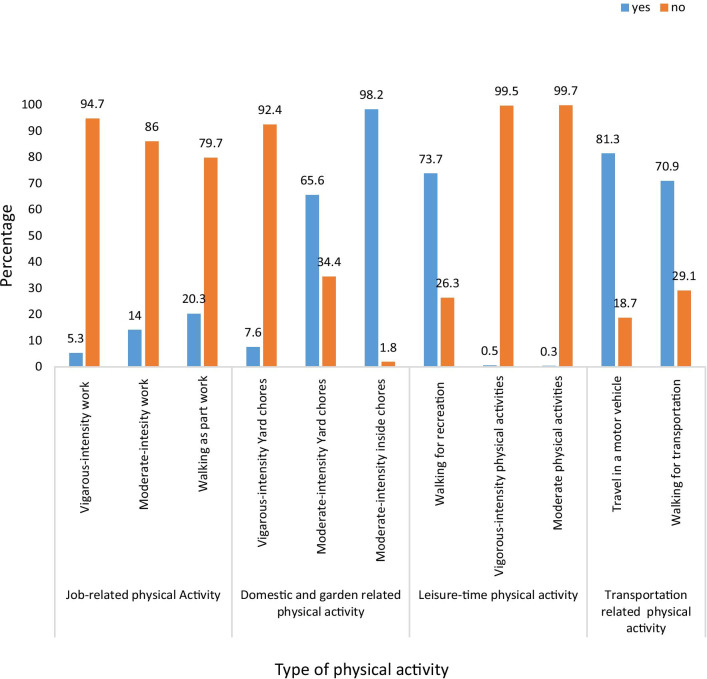
Proportion of women who engage in physical activity by specific domains and intensity level in Addis Ababa, Ethiopia, 2019

Of the total study participants, 5.3% reported doing vigorous-intensity physical activity that lasted for at least 10-minutes continuously while at work. Whereas 14% reported doing moderate-intensity physical activity for at least 10-minutes continuously while at work. Moderate-intensity inside chores and activities were the most commonly practiced physical activities (98.2%) among women. Vigorous- and moderate-intensity leisure-time activities were the least commonly practiced among women (Fig. 2).

The MET minutes per week (median (interquartile range)) of the women were 742 (330, 2145) for job related physical activities; 360 (180, 630) for domestic and garden work; 231 (132, 346) for recreation and leisure related physical activities; and 231 (132, 396) for transportation related physical activities. The proportion of women who engaged in a high, moderate and low level of physical activity was 10.9%; 42.0% and 47.1%, respectively.

### Intimate partner violence and perinatal depression

Of the total respondents, 18.2% experienced intimate partner violence (any physical, sexual or emotional violence) during their current pregnancy; 6.6% experienced physical violence; 7.3% sexual violence; and 8.1% emotional violence. Forty one percent (41%) asked permission from their partners to seek health care. Ten percent (10%) of the respondents had symptoms related to perinatal depression.

### Predictors of gestational weight gain

Seventy seven percent (77%) of women who did not attend formal education gained inadequate gestational weight, while 56% of women who attended tertiary education gained inadequate gestational weight. Seventy six percent (76%) of women with no paid employment outside of home (home duties) gained inadequate gestational weight, however 55% of women who were employed by a government or private institution gained inadequate weight. Fourteen percent (14%) of women with perinatal depression gained excessive gestational weight compared to women with no perinatal depression (4%).

A multivariable regression model included: women’s educational status, occupation, wealth index, pre-pregnancy BMI, maternal mid-upper arm circumference, perinatal depression and whether the women had asked permission from their partners to seek health care. Compared to overweight or obese women, being underweight (AOR = 3.30 [95% CI: 1.32, 8.24]) and normal weight (AOR = 2.68 [95% CI: 1.37, 5.24]) increased the odds of gaining inadequate gestational weight. Similarly, Not having paid employment, (AOR = 2.17 [95% CI: 1.16, 4.07]), was associated with higher odds of gaining inadequate gestational weight compared to women employed by the government or private institutions. The odds of inadequate GWG in the second lowest wealth quartile was 61% less than in lowest wealth quartile women (Table 4).

**Table 4:** Predictors of gestational weight gain in Addis Ababa, Ethiopia, 2019

	Proportions of GWG			Inadequate vs Adequate			Excess vs Adequate		
Variable	Inadequate GWG n(%)	Adequate GWG n(%)	Excess GWG n(%)	Crude Odds Ratio (COR)	Adjusted Odds Ratio (AOR)	*P*-value for AOR	Crude Odds Ratio (COR)	Adjusted Odds Ratio (AOR)	*P*-value for AOR
Educational status	*P*-value = 0.184								
No formal education	27 (77.1)	7 (20.0)	1(2.9)	2.69 [1.04, 6.98]	2.16 [0.70, 6.66]	0.178	1.07 [0.10, 11.13]	1.20 [0.09, 16.86]	0.894
Primary education (1–8)	100 (65.8)	44 (28.9)	8(5.3)	1.59 [0.88, 2.85]	1.34 [0.63, 2.84]	0.442	1.36 [0.38, 4.94]	1.60 [0.32, 7.83]	0.569
Secondary education (9–12)	77 (74.0)	22 (21.2)	5 (4.8)	2.44 [1.26, 4.75]	1.63 [0.74, 3.60]	0.159	1.70 [0.41, 7.09]	1.66 [0.33, 8.41]	0.542
Tertiary education	43 (55.8)	30 (39.0)	4 (5.2)	Reference	Reference		Reference	Reference	
Wealth quartile	*P*-value = 0.247								
Lowest	53 (74.7)	15 (21.1)	3 (4.2)	Reference	Reference		Reference	Reference	
Second	45 (59.2)	30 (39.5)	1 (1.3)	0.42 [0.20, 0.89]	0.39 [0.17, 0.86]	0.021	0.17 [0.02, 1.74]	0.09 [0.01, 1.10]	0.060
Middle	53 (70.7)	18 (24.0)	4 (5.3)	0.83 [0.38, 1.82]	0.91 [0.39, 2.12]	0.832	1.11 [0.21, 5.76]	1.32 [0.22, 7.94]	0.762
Fourth	53 (69.7)	18 (23.7)	5 (6.6)	0.83 [0.38, 1.82]	1.12 [0.46, 2.72]	0.806	1.39 [0.28, 6.79]	1.95 [0.33, 11.46]	0.459
Highest	44 (62.0)	22 (31.0)	5 (7.0)	0.57 [0.26, 1.22]	0.83 [0.34, 2.01]	0.679	1.14 [0.24, 5.49]	1.63 [0.27, 9.93]	0.598
Occupational status	*P*-value = 0.015								
Employee	66 (55.5)	46 (38.7)	7 (5.9)	Reference	Reference		Reference	Reference	
Do not have paid employment	140(76.1)	37 (20.1)	7 (3.8)	2.64 [1.56, 4.45]	2.17[1.16, 4.07]	0.016	1.24 [0.40, 3.86]	1.00 [0.26, 3.83]	0.998
Merchant	26 (66.7)	10 (25.6)	3 (7.7)	1.81 [0.80, 4.12]	2.14 [0.81, 5.61]	0.123	1.97 [0.43, 8.93]	1.58 [0.26, 9.68]	0.623
Other	16 (59.3)	10 (37.0)	1 (3.7)	1.12 [0.46, 2.68]	0.76 [0.29, 2.01]	0.585	0.66 [0.07, 5.95]	0.65 [0.06, 6.92]	0.723
Weight status									
Underweight	51 (75.0)	15 (22.1)	2 (2.9)	3.52 [1.62, 7.65]	3.30 [1.32, 8.24]	0.011	0.48 [0.09, 2.57]	0.25 [0.03, 1.97]	0.188
N ormal weight	169 (71.6)	59 (25.0)	8 (3.4)	2.97 [1.63, 5.39]	2.68 [1.37, 5.24]	0.004	0.49 [0.17, 1.44]	0.39 [0.11, 1.37]	0.141
Overweight or obese	28 (43.1)	29 (44.6)	8 (12.3)	Reference	Reference		Reference	Reference	
MUAC	P-value = 0.187								
Low MUAC (< 23 cm)	49 (75.4)	12 (18.5)	4 (6.1)	1.85 [0.94, 3.63]	1.94 [0.87, 4.32]	0.103	2.10 [0.59, 7.42]	6.63 [1.38, 31.89]	0.018
High MUAC (≥ 23 cm)	194 (65.6)	88 (29.7)	14 (4.7)	Reference	Reference		Reference	Reference	
Perinatal depression	*P*-value = 0.015								
Yes	26 (72.2)	5(13.9)	5 (13.9)	2.30 [0.86, 6.15]	2.56 [0.88, 7.46]	0.086	7.54 [1.92, 29.61]	12.50 [2.72, 57.54]	0.001
No	222 (66.7)	98 (29.4)	13 (3.9)	Reference	Reference		Reference	Reference	
Asking permission from partners for health care seeking	*P*-value = 0.123								
Yes	112 (73.2)	34 (22.2)	7 (4.6)	1.65 [1.02, 2.67]	1.44 [0.83, 2.50]	0.191	1.27 [0.45, 3.58]	1.98 [0.59, 6.70]	0.270
No	136 (63.3)	68 (31.6)	11 (5.1)	Reference	Reference		Reference	Reference	
Age	*P*-value = 0.965								
Less than 20 years	12 (66. 7)	6 (33.3)	0	0.83 [0.30, 2.28]	NA	NA	–	NA	NA
20–29 years	198 (66.7)	82 (27.6)	17 (5.7)	Reference	NA	NA	Reference	NA	NA
30–40 years	38 (70.4)	15 (27.8)	1 (1.8)	1.05 [0.55, 2.01]	NA	NA	0.32 [0.04, 2.60]	NA	NA
Partners’ educational status (369)	*P*-value = 0.782								
No formal education	25 (83.3)	5(16.7)	0	2.43 [0.86, 6.90]	NA	NA	–	NA	NA
Primary education (1–8)	62 (65.3)	27 (28.4)	6 (6.3)	1.12 [0.61, 2.05]	NA	NA	1.26 [0.36, 4.35]	NA	NA
Secondary education (9–12)	91 (67.9)	37 (27.6)	6 (4.5)	1.19 [0.68, 2.09]	NA	NA	0.92 [0.27, 3.12]	NA	NA
Tertiary education	70 (63.6)	34 (30.9)	6(5.5)	Reference		NA	Reference	Reference	
Minimum dietary diversity	*P*-value = 0.851								
Low dietary diversity	47 (70.1)	17 (25.4)	3 (4.5)	1.18 [0.64, 2.18]	NA	NA	1.01[0.26, 3.88]	NA	NA
High dietary diversity	201(66.5)	86 (28.5)	15 (5.0)	Reference	NA	NA	Reference	NA	NA
Food insecurity	*P*-value = 0.903								
Food secure	212 (67.1)	89 (28.2)	15 (4.7)	Reference	NA	NA	Reference	NA	NA
Food insecure	33 (67.4)	13 (26.5)	3 (6.1)	1.07 [0.54, 2.12]	NA	NA	1.37 [0.35, 5.39]	NA	NA
Intimate partner violence	*P*-value = 0.335								
Yes	44 (62.8)	24 (34.3)	2 (2.9)	0.71 [0.41, 1.24]	NA	NA	0.41 [0.09, 1.92]	NA	NA
No	204 (68.2)	79 (26.4)	16 (5.4)	Reference	NA	NA	Reference	NA	NA
Type of pregnancy (363)	*P*-value = 0.990								
Intended	221(67.0)	91 (27.6)	18(5.4.)	Reference	NA	NA	Reference	NA	NA
Unintended	23 (69.7)	10 (30.3)	0	0.95 [0.43, 2.07]	NA	NA	–	NA	NA
Number of pregnancy (369)	*P*-value = 0.333								
First pregnancy	116 (64.8)	55 (30.7)	8 (4.5)	Reference	NA	NA	Reference	NA	NA
Second	80 (74.1)	25 (23.1)	3 (2.8)	1.52 [0.87, 2.63]	NA	NA	0.83 [0.20, 3.37]	NA	NA
Third	36 (67.9)	13 (24.5)	4 (7.6)	1.31 [0.63, 2.67]	NA	NA	2.12 [0.55, 8.12]	NA	NA
Fourth and above	16 (55.2)	10 (34.5)	3(10.3)	0.76[0.32, 1.78]	NA	NA	2.06 [0.47, 9.13]	NA	NA
Level of physical activity (369)	*P*-value = 0.633								
Low	120 (68.6)	47 (26.8)	8 (4.6)	1.60 [0.77, 3.30]	NA	NA	2.55 [0.29, 22.10]	NA	NA
Moderate	104 (67.5)	41 (26.6)	9 (5.9)	1.59 [0.76, 3.32]	NA	NA	3.29 [0.38, 28.24]	NA	NA
High	24 (60.0)	15 (37.5)	1 (2.5)	Reference	NA	NA	Reference	NA	NA

NA* not illegible to be included into multivariable regression because the *p*-values were > 0.25 in bi-variable regression

## Discussion

We found that more than two thirds of women (67.2%) gained inadequate gestational weight. In contrast, fewer than 5% of women gained excess gestational weight. Being underweight or normal weight before pregnancy increased the odds of gaining inadequate gestational weight. Similarly, not having paid employment (home duties) was associated with higher odds of gaining inadequate gestational weight compared to women employed by government or private institutions. The odds of inadequate GWG for women in the second lowest wealth quartile was 61% less than women in lowest wealth quartile.

Inadequate gestational weight gain was high in our study, with more than two-thirds of women gaining inadequate gestational weight. This may be due to some women in Ethiopia decreasing food consumption during pregnancy fearing that overeating may enlarge the fetal head or make the baby overweight and that will lead to a caesarean birth [59, 60]. Wang et al. recently used data from the demographic health survey program to estimate the average levels of GWG in all low and middle income countries (LMIC) and found that the average level of GWG is lower than the United State Institute of Medicine recommendations in most LMIC countries [13]. Our findings are consistent, however, with other studies in sub-Saharan Africa, including the proportions of women with inadequate gestational weight gain of 71.8% in Malawi [61], 63% in Niger [62], and 62.7% in Uganda [14]. A higher proportion of women with inadequate GWG in these low-income sub-Saharan Africa settings, including Ethiopia, could be as a result of a wide range of nutritional problems, economic instability, poverty, food insecurity, and frequent infections which are common in sub-Saharan Africa [63]. In addition, a significant proportion of women were underweight at the time of conception in sub-Saharan Africa [5, 12, 64]. Moreover, more than a half of pregnant women in sub-Saharan African settings lack awareness of the risks associated with inappropriate GWG [65]. Given the adverse maternal and child health outcomes associated with inadequate GWG [43, 66–68], our findings indicate that inadequate GWG is a public health concern in Addis Ababa, Ethiopia and highlights the need for effective maternal health interventions to influence weight gain during pregnancy.

Our findings contrast with high-income countries where a higher proportion of women with excessive GWG have been reported, where the majority of women experience excessive GWG [2, 6, 7]. Adequate gestational weight gain was found in fewer than one-third (27.9%) of our study participants, which is similar to many other studies [2–11]. This would indicate that the practical applicability of the IOM guideline needs further investigation. The IOM guidelines are explicitly intended as recommendations for women in the United States which may be applicable for women in other high-income countries. The IOM guidelines were primarily intended to prevent excessive GWG. However, previous studies conducted in LMIC have used the IOM guidelines to facilitate comparisons across countries, as there are no specific guidelines for gestational weight gain for LMIC. This highlights the urgent need for GWG recommendations dedicated to LMIC or consideration of data from LMIC.

The prevalence of inadequate GWG was 75% among underweight women, compared with 43% among overweight or obese women. Studies from sub-Saharan African countries have also reported that more than three-quarters of women who were underweight at the conception of the pregnancy gain gestational weight below the IOM recommendations [5, 64]. In our study, being underweight increased the odds of gaining inadequate gestational weight three-fold, while being normal weight increased the odds of gaining inadequate gestational weight two-fold, compared to overweight or obese women. This is due to the fact that pre-pregnancy BMI is closely linked to maternal nutrition, lifestyle and socio-cultural factors, which could have an impact on the amount of GWG [69]. Women who are underweight or normal weight before pregnancy are required to gain more weight than their overweight or obese counterparts to achieve a healthy GWG. Overweight and obese women, on the other hand, are required to gain comparatively little weight to achieve adequate GWG as they are able to use a portion of their stored energy to support the growth of the fetus. As such, adequate GWG may be attained easily for these women. This would potentially provide further evidence for strategies to support the promotion of adequate gestational weight gain in Ethiopian underweight and normal weight women.

We identified that not being in paid employment was associated with higher odds of inadequate GWG compared to women employed outside the home by government or private institutions. This could be due to half of our study participants not having paid employment at the time of data collection. This high proportion of women’s unemployment is associated with women’s educational status. About half of our study participants attended no formal education or only attended primary education. Maternal education would help women to generate income through employment, as educated women are more likely to have paid work than uneducated women [70]. A study from China also reported that unemployment, housework or temporary work increased the odds of inadequate GWG compared to regular paid employment outside the home [71]. Improving women’s employment status would be important to prevent inappropriate GWG.

While studies from high-income countries reported that household food insecurity is associated with excessive GWG [32, 33], household food insecurity was not significantly associated with GWG in our study. Women’s dietary diversity, intimate partner violence and physical activity were not significantly associated with GWG.

Previous studies have reported that perinatal depression is associated with gaining inadequate gestational weight [25] or excessive GWG [72, 73], while another study reported perinatal depression was not associated with either inadequate or excessive GWG [74]. In our study, perinatal depression was not associated with inadequate GWG, but associated with excessive GWG. However, since few women in our study gained excessive gestational weight and those findings had a wide confidence interval, this is not strong evidence to conclude that perinatal depression is associated with excessive GWG.

About half of our study participants (47.1%) reported that they engaged in low-level physical activity. Physical activity was not significantly associated with GWG in our study. The women in our study used the highest amount of energy on household activities. This could be due to 50% of our study participants being unemployed (identified themselves as undertaking home duties), which could have accounted for the low energy expenditure on occupational activity. Another possible reason could be most pregnant women may feel safer and comfortable doing household activities than engaging in occupational or sports activities during pregnancy [75].

This study has some limitations. Firstly, the IOM GWG recommendations are the recommendations of high-income countries. These recommendations may not be suitable in low-income settings such as Ethiopia. Secondly, we measured pre-pregnancy BMI before or at 16 weeks of gestation, at which time there may already have been an increase or decrease of gestational weight. In addition, women’s last weight was measured at 36 weeks of gestation; hence there may be some weight gain after 36 weeks of gestation. Finally, this study was conducted in the capital city of Ethiopia in the public health facilities; the situation in other parts of the country and private health facilities may be different. Future research may need to investigate the effect of GWG on pregnancy outcomes.

## Conclusion

Nearly two-thirds of our study participants gained inadequate gestational weight. Pre-pregnancy BMI and occupational status of the women were strong predictors of GWG. Preconception or early pregnancy GWG-related counselling and intervention(s) regarding the best GWG management approach would be a public health priority. The practical applicability of the IOM guidelines and the effect of GWG (according to IOM recommendations) on pregnancy outcomes need further investigation in Ethiopian context.

## Supplementary Information

**Additional file 1. Table S1:** Number of women participated from different health centres, Addis Ababa, Ethiopia, 2019

## Data Availability

The datasets used for the analysis are available from the corresponding author on reasonable request.

## Abbreviations

ANC: Antenatal Care
AOR: Adjusted Odds Ratio
BMI: Body Mass Index
EPDS: Edinburgh Postnatal Depression Scale
FAO: Food and Agricultural Organisation
GWG: Gestational Weight Gain
HFIAS: Household Food Insecurity Access Scale
IOM: Institute of Medicine
IQR: Inter Quartile Range
IPAQ: International Physical Activity Questionnaire
LBW: Low Birth Weight
MDD-W: Women’s-Minimum Dietary Diversity
MET: Metabolic Equivalent Task
MUAC: Mid-Upper Arm Circumferences
USAID: United States Agency for International Development
