# Supplementary material for: Patterns and predictors of gestational weight gain in Addis Ababa, Central Ethiopia: a prospective cohort study
Source: Reprod Health. 2021 Jul 28;18:159. doi: 10.1186/s12978-021-01202-y (PMC8317358; doi:10.1186/s12978-021-01202-y)
Supplement: Supplementary file 1 — Additional file 1. Table S1: Number of women participated from different health centres, Addis Ababa, Ethiopia, 2019 [file 12978_2021_1202_MOESM1_ESM.docx]

Additional file 1: Table S1: Number of women participated from different health centres, Addis Ababa, Ethiopia, 2019

| Name of health centre | Number of participating women | Percentage |
| --- | --- | --- |
| Milililand | 34 | 8.6 |
| Kolfe | 81 | 20.5 |
| Keraniyo | 37 | 9.4 |
| Woerda 09 Kowas Meda | 28 | 7.1 |
| Alembank | 47 | 11.9 |
| Kotobe | 110 | 27.9 |
| Woreda 11 of Nifas Silk lafto Sub city | 8 | 2.0 |
| Woreda 02 of Nifas Silk lafto Sub city | 42 | 10.6 |
| Woreda 05 of Nifas Silk lafto Sub city | 8 | 2.0 |
| Total | 395 | 100 |
